# Causal Inference Approaches Reveal Associations Between LDL Oxidation, NO Metabolism, Telomere Length and DNA Integrity Within the MARK-AGE Study

**DOI:** 10.3390/antiox14080933

**Published:** 2025-07-30

**Authors:** Andrei Valeanu, Denisa Margina, María Moreno-Villanueva, María Blasco, Ewa Sikora, Grazyna Mosieniak, Miriam Capri, Nicolle Breusing, Jürgen Bernhardt, Christiane Schön, Olivier Toussaint, Florence Debacq-Chainiaux, Beatrix Grubeck-Loebenstein, Birgit Weinberger, Simone Fiegl, Efstathios S. Gonos, Antti Hervonen, Eline P. Slagboom, Anton de Craen, Martijn E. T. Dollé, Eugène H. J. M. Jansen, Eugenio Mocchegiani, Robertina Giacconi, Francesco Piacenza, Marco Malavolta, Daniela Weber, Wolfgang Stuetz, Tilman Grune, Claudio Franceschi, Alexander Bürkle, Daniela Gradinaru

**Affiliations:** 1 Faculty of Pharmacy, Carol Davila University of Medicine and Pharmacy, 6 Traian Vuia Street, 020956 Bucharest, Romania; andrei.valeanu@umfcd.ro (A.V.); denisa.margina@umfcd.ro (D.M.); 2 Molecular Toxicology Group, Department of Biology, University of Konstanz, 78457 Konstanz, Germany; maria.moreno-villanueva@uni-konstanz.de (M.M.-V.); alexander.buerkle@uni-konstanz.de (A.B.); 3 Human Performance Research Centre, Department of Sport Science, University of Konstanz, 78457 Konstanz, Germany; 4 Spanish National Cancer Research Centre (CNIO), 28029 Madrid, Spain; mblasco@cnio.es; 5 Laboratory of the Molecular Bases of Ageing, Nencki Institute of Experimental Biology, Polish Academy of Sciences, 3 Pasteur Street, 02-093 Warsaw, Poland; e.sikora@nencki.edu.pl (E.S.); g.mosieniak@nencki.edu.pl (G.M.); 6 Department of Medical and Surgical Sciences (DIMEC), Alma Mater Studiorum, University of Bologna, 40126 Bologna, Italy; miriam.capri@unibo.it (M.C.); claudio.franceschi@unibo.it (C.F.); 7 Alma Mater Research Institute on Global Challenges and Climate Change (Alma Climate), University of Bologna, 40126 Bologna, Italy; 8 Department of Applied Nutritional Science/Dietetics, Institute of Nutritional Medicine, University of Hohenheim, 70599 Stuttgart, Germany; nicolle_breusing@yahoo.de; 9 BioTeSys GmbH, Schelztorstr. 54-56, 73728 Esslingen, Germany; j.bernhardt@biotesys.de (J.B.); c.schoen@biotesys.de (C.S.); 10 Research Unit on Cellular Biology, University of Namur (URBC-NARILIS), University of Namur, Rue de Bruxelles, 61, 5000 Namur, Belgiumflorence.chainiaux@unamur.be (F.D.-C.); 11 Research Institute for Biomedical Aging Research, Universität Innsbruck, Rennweg, 10, 6020 Innsbruck, Austria; beatrix.grubeck-loebenstein@uibk.ac.at (B.G.-L.); birgit.weinberger@uibk.ac.at (B.W.); 12 Institute for Nutritional Sciences and Physiology, Private University for Health Sciences, Medical Informatics and Technology (UMIT TIROL), Eduard-Wallnöfer-Zentrum 1, 6060 Hall in Tyrol, Austria; simone.fiegl@umit-tirol.at; 13 National Hellenic Research Foundation, Institute of Biology, Medicinal Chemistry and Biotechnology, 116 35 Athens, Greece; sgonos@eie.gr; 14 Medical School, University of Tampere, 33014 Tampere, Finland; antti.hervonen@gmail.com; 15 Section of Molecular Epidemiology, Leiden University Medical Centre, 2333 ZG Leiden, The Netherlands; p.slagboom@lumc.nl; 16 Department of Gerontology and Geriatrics, Leiden University Medical Center, 2333 ZG Leiden, The Netherlands; 17 Centre for Health Protection, National Institute for Public Health and the Environment, P.O. Box 1, 3720 BA Bilthoven, The Netherlands; martijn.dolle@rivm.nl (M.E.T.D.); eugenejansen99@gmail.com (E.H.J.M.J.); 18 Advanced Technology Center for Aging Research and Geriatric Mouse Clinic, IRCCS INRCA, 60121 Ancona, Italy; e.mocchegiani@inrca.it (E.M.); r.giacconi@inrca.it (R.G.); f.piacenza@inrca.it (F.P.); m.malavolta@inrca.it (M.M.); 19 Department of Clinical and Molecular Sciences (DISCLIMO), Università Politecnica delle Marche, 60126 Ancona, Italy; 20 Department of Molecular Toxicology, German Institute of Human Nutrition, Potsdam-Rehbrücke, 14558 Nuthetal, Germany; daniela.weber@dife.de (D.W.); scientific.director@dife.de (T.G.); 21 NutriAct-Competence Cluster Nutrition Research Berlin-Potsdam, 14458 Nuthetal, Germany; 22 Department of Food Biofunctionality, Institute of Nutritional Sciences (140), University of Hohenheim, 70599 Stuttgart, Germany; wolfgang.stuetz@uni-hohenheim.de; 23 German Center for Diabetes Research (DZD), 85764 Oberschleißheim, Germany; 24 German Center for Cardiovascular Research (DZHK), Partner Site Berlin, 13347 Berlin, Germany; 25 Institute of Nutritional Science, University of Potsdam, 14458 Nuthetal, Germany; 26 Department of Physiological Chemistry, Faculty of Chemistry, University of Vienna, 1090 Vienna, Austria; 27 Laboratory of Systems Medicine of Healthy Aging, Institute of Biology and Biomedicine and Institute of Information Technology, Mathematics and Mechanics, Department of Applied Mathematics, N. I. Lobachevsky State University, Nizhny Novgorod 603005, Russia; 28 Ana Aslan National Institute of Gerontology and Geriatrics, 11241 Bucharest, Romania

**Keywords:** genomic instability markers, LDL oxidation, nitric oxide, causal analysis, MARK-AGE, vascular aging

## Abstract

Genomic instability markers are important hallmarks of aging, as previously evidenced within the European study of biomarkers of human aging, MARK-AGE; however, establishing the specific metabolic determinants of vascular aging is challenging. The objective of the present study was to evaluate the impact of the susceptibility to oxidation of serum LDL particles (LDLox) and the plasma metabolization products of nitric oxide (NOx) on relevant genomic instability markers. The analysis was performed on a MARK-AGE cohort of 1326 subjects (635 men and 691 women, 35–75 years old) randomly recruited from the general population. The Inverse Probability of Treatment Weighting causal inference algorithm was implemented in order to assess the potential causal relationship between the LDLox and NOx octile-based thresholds and three genomic instability markers measured in mononuclear leukocytes: the percentage of telomeres shorter than 3 kb, the initial DNA integrity, and the DNA damage after irradiation with 3.8 Gy. The results showed statistically significant telomere shortening for LDLox, while NOx yielded a significant impact on DNA integrity. Overall, the effect on the genomic instability markers was higher than for the confirmed vascular aging determinants, such as low HDL cholesterol levels, indicating a meaningful impact even for small changes in LDLox and NOx values.

## 1. Introduction

Establishing the specific biomarkers of vascular endothelium function is important for the complex evaluation of biological age in humans. According to Sir William Osler (1891), “*Longevity is a vascular question which has been well expressed in the axiom that man is only as old as his arteries*” [1]. This old axiom has been widely confirmed by epidemiological and observational studies establishing that vasculature aging is a complex process that in a large measure reflects the overall aging of the individual [2,3]. Although aging is a major risk factor for the incidence and progression of a wide range of pathologies, drawing a clear line between physiological and pathological aging using cellular and systemic specific biomarkers is still challenging. In this regard, at the systemic level, LDL oxidizability (LDLox) and nitric oxide metabolic pathway products (NOx) are currently recognized as representative biomarkers of oxidative stress, inflammation, and endothelial dysfunction, and are being assessed within the EU project MARK-AGE as candidate biomarkers of aging and cardiometabolic disease risk [4,5].

At the cellular level, it has been demonstrated that DNA repair capacity and the maintenance of genomic stability are both intimately linked to the aging process; therefore, relevant specific biomarkers were included in the MARK-AGE study [6]. Several older and more recent studies prove that telomere length, usually measured from the white blood cells, can be considered a marker of aging and of general health status [3,7,8,9,10]. Telomere shortening, which occurs due to the end-replication problem and reduced telomerase activity, is an important predictor of biological aging and can be accelerated by oxidative DNA damage [8,9]. Furthermore, critically short telomeres modify the immediate cellular response to DNA damage generated by endogenous and exogenous damaging agents, which can reduce DNA integrity [11].

With regard to the relationship between LDL oxidation (and NO metabolization products—NOx) and telomere length or DNA integrity, in vivo oxidized LDL (oxLDL) levels have been shown to be inversely correlated with telomere length in both smokers and non-smokers, after adjusting for age and sex [3]. In addition, oxLDL has been shown to induce oxidative DNA damage, while nitric oxide can sensitize the cells to ionizing radiation [12,13]. Nevertheless, no study has specifically analyzed the possible impact of NOx on telomere length, nor has any research evaluated in more detail, within a large population study or by means of multi-variable adjustment or machine learning approaches, the impact of LDL oxidation on these genomic instability markers or on the related DNA damage processes.

The MARK-AGE was an important population study that identified a specific combination of biomarkers that could explain biological age, including telomere length [6]. However, the systemic drivers of cellular telomere shortening and DNA damage through oxidative stress and other specific mechanisms have largely remained unexplored with regard to MARK-AGE data analysis, while machine learning approaches to such data are still scarce or focused rather on general machine learning recommendations or predictive modelling of cardiometabolic risk [5,14].

Therefore, given the scarcity of studies regarding the interrelationship between LDL oxidation, NOx, and telomere shortening or DNA integrity, as well as the complex information collected within the MARK-AGE dataset, the aim of the current research was to estimate the impact of LDL oxidizability and NO metabolization products on telomere length and DNA integrity by means of causal inference approaches.

## 2. Materials and Methods

### 2.1. Study Design

This cross-sectional observational study was carried out on a relevant population sample of 1326 subjects, comprising 635 men and 691 women, aged between 35 and 75 years old, and selected from among the participants included in the MARK-AGE group of randomly recruited age- and sex-stratified individuals from the general population (RASIG). Only MARK-AGE subjects with complete data for all studied parameters were included in the present study. Participants from the MARK-AGE cohort were enrolled, through the media, from seven European countries: Austria, Belgium, Finland, Germany, Greece, Italy, and Poland. Subjects who reported seropositivity for HIV or hepatitis (HBV and HCV), whose blood was tested positive for HBV or HCV, or who were being treated for cancer or receiving glucocorticoids were excluded from the study [5,6].

The biological samples (fasting blood) collected from participants were processed and stored within the MARK-AGE consortium, according to rigorous Standard Operating Procedures and quality control measures, as described [5,6]. Briefly, the double-coded blood samples (plasma and serum) were centrally stored in a biobank and distributed to each MARK-AGE partner for the independent measurement of the specific candidate biomarkers. All of the subjects’ clinical and biochemical data obtained from each partner were uploaded to a central database that also contained the demographic and anthropometric data. This phenotypic database could only be accessed and analyzed at the end of the MARK-AGE project [6,14].

The following parameters were selected from the central database and considered for the present analysis: serum glucose and insulin; cholesterol of the serum lipoprotein fractions LDL and HDL; LDL oxidation susceptibility (LDL oxidizability, LDLox); plasma-stable metabolic pathway products of NO (NOx); plasma tocopherols (α-/γ-tocopherol), carotenoids (α-/β-carotene, lycopene), retinol, and vitamin D; plasma iron (Fe), selenium (Se), copper (Cu), and zinc (Zn); DNA integrity and telomere length, assessed in peripheral blood mononuclear cells (PBMCs).

Standard demographic (age, sex) and anthropometric data (height, weight, waist circumference, body mass index, waist-to-hip ratio) were obtained from each participant. The resting systolic and diastolic blood pressures (SBP and DBP, mmHg) were recorded for all subjects, as well as whether there was currently a diagnosed high blood pressure problem. Participants completed a comprehensive questionnaire that included information on self-reported past and present diseases, hormone therapy (women), self-rated health status, and the following lifestyle characteristics: smoking status (never, former, or current smoker); number of years of smoking (smoking years); alcohol and other beverage consumption (whether the subjects never consume beer, wine, juice, or cola beverages); nutritional status (the quantitative consumption of meat, fish, eggs, bread, rice, fruits, vegetables, salty snacks, and sweets); educational background; marital status; information about residence, i.e., house or apartment and whether the subjects live with children, other relatives, or friends [5,6].

### 2.2. Laboratory Methods

Serum glucose, LDL cholesterol and HDL cholesterol were measured on the clinical auto-analyzer (LX20-Pro, Beckman–Coulter, Woerden, The Netherlands). Insulin was measured with an immuno-analyzer (Access-2, Beckman–Coulter, Woerden, The Netherlands).

Insulin resistance was evaluated using the Homeostasis Model Assessment—Insulin Resistance (HOMA-IR) as surrogate markers, and calculated as follows: [fasting insulin (mU/L) × fasting glucose (mg/dL)]/405 [15].

LDL oxidation susceptibility (LDL oxidizability, LDLox) was assessed in vitro using serum LDL isolated through selective precipitation and the measurement of the specific products of the lipid peroxidation chain reaction, after the exposure of LDL particles to a standard oxidative stress inducer, as previously described [5]. The results were expressed as nmol malondialdehyde (MDA) equivalent content/mL serum. The intra-assay CV was 6.5% and the inter-assay CV was 7.4%.

The total amount of plasma-stable metabolic pathway products of NO [NOx, the sum of nitrites and nitrates (NO_2_^−^ + NO_3_^−^)] was determined using the Griess reagent, following the quantitative conversion of nitrates (NO_3_^−^) to nitrites (NO_2_^−^) with nitrate reductase (kit 23479, SIGMA). The results were expressed in μmols NOx/L plasma. Intra- and inter-assay CVs were below 7% and 9%, respectively.

Tocopherols (α-/γ-tocopherol), carotenoids (α-/β-carotene, lycopene), and retinol in plasma were simultaneously determined by HPLC and spectrophotometric and fluorescence detection as previously described [16].

Serum vitamin D was measured with an enzyme immunoassay (EIA): 25OHVitD (OCTEIA, AC57F1, IDS, Boldon, UK).

Plasma iron (Fe), selenium (Se), copper (Cu), and zinc (Zn) were determined using a Thermo XII Series ICP-MS (Thermo Electron Corporation, Waltham, MA, USA) according to previously described methods used for the measurement of trace elements in human plasma [17].

The DNA damage and the repair of DNA strand breaks (DNA integrity) were measured using a modified and automated version of the Fluorimetric Detection of Alkaline DNA Unwinding [18]. Cryopreserved isolated peripheral blood mononuclear cells (PBMCs) were immediately irradiated with a 3.8 Gy irradiation dose and incubated at 37 °C for 40 min to allow DNA repair.

The telomere length was determined in isolated PBMCs using an automated high-throughput quantitative fluorescence in situ hybridization (HT Q-FISH) telomere length analysis platform [19]. HT Q-FISH combines the labelling of telomeres in interphase nuclei, using a fluorescent peptide nucleic acid (PNA) probe against telomeric repeats, with automated HT microscopy in 96-well plates. The low detection limit of Q-FISH (<0.1 kb of telomere repeats) allows the quantification of critically short telomeres, the frequency of which is a determinant of telomere dysfunction.

### 2.3. Statistical Analysis

#### 2.3.1. General Statistical Analysis—Causal Algorithm

The main aim of the statistical analysis was to estimate the impact of candidate vascular aging markers, namely LDLox and NOx, on telomere length and DNA integrity as well-established biomarkers with a critical impact on aging. The analysis was undertaken in Python Programming Language, version 3.9.2 [20]. In order to obtain a less biased effect estimate, a causal inference algorithm was used, namely the Inverse Probability of Treatment Weighting (IPTW) from the DoWhy package, which can take into account imbalanced binary interventions [21].

For each IPTW implementation, specific threshold values were used for LDLox and NOx, as well as for a narrow group of relevant biomarkers for the metabolic profile, cardiometabolic risk, and vascular aging, namely LDL cholesterol and HDL cholesterol, blood pressure (SBP and DBP), and HOMA-IR, respectively, which were considered as “interventions”. The percentage of telomeres shorter than 3 kb, the initial DNA integrity, and the DNA damage after irradiation with 3.8 Gy were the three “continuous outcomes”. The IPTW algorithm estimated the effect of each binary intervention on each continuous outcome [21].

#### 2.3.2. Setting the Thresholds for the Intervention Variables

The thresholds were created based on the information from the updated clinical guidelines for HDL-C, LDL-C, blood pressure, and HOMA-IR [22,23,24,25], while for LDLox and NOx they were set based upon splitting the dataset into octiles (Q12.5; Q25; Q37.5; Q50; Q62.5; Q75; and Q87.5), since no standardized risk values are yet recommended for these two biomarkers. In addition to the seven octiles for LDLox and NOx, seven combined thresholds between each octile of LDLox and NOx were created (Risk_NOx+LDLox_Q12.5; Q25; Q37.5; Q50; Q62.5; Q75; and Q87.5, respectively, representing the concomitant status of LDLox and NOx above the specific octile based thresholds) in order to account for the cumulative impact of LDLox and NOx. Table 1 summarizes the threshold values, as well as the percentual frequency (% of subjects) for each threshold. Therefore, it should be mentioned that the IPTW algorithm was applied for each of the three outcomes and each threshold value: in each case, it estimated the increase (or decrease) in the outcome value when the specific marker value was above the mentioned threshold (or below the threshold, according to the mentioned sign), while also adjusting for specific confounders.

#### 2.3.3. Setting the Confounding Variables and Validating the Estimated Effects

**Figure 1:**
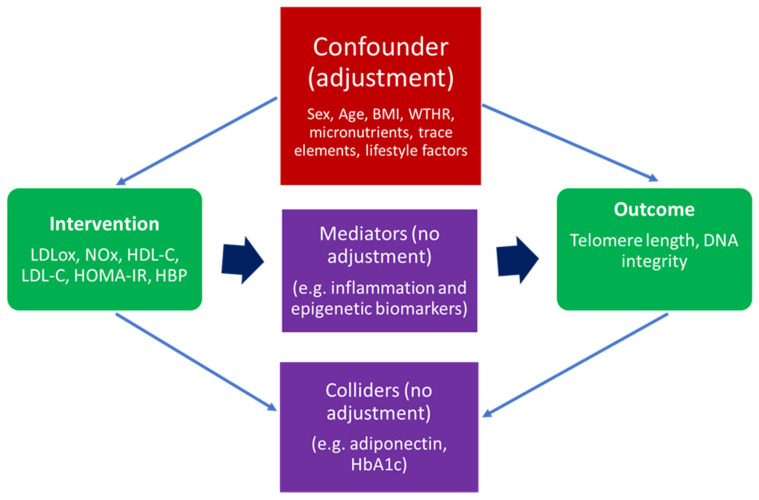
A general causal diagram representing MARK-AGE parameters included in IPTW algorithm as an intervention, outcome confounder, mediator, and collider. Legend: BMI, body mass index; WTHR, waist-to-hip ratio; LDLox, low-density lipoprotein susceptibility to oxidation; NOx, nitric oxide metabolic pathway products; LDL-C, LDL cholesterol; HDL-C, HDL cholesterol; HOMA-IR, Homeostasis Model Assessment—Insulin Resistance; HbA1C, glycosylated hemoglobin A1C; HBP, high blood pressure.

An essential step for the implementation of the IPTW algorithm was the careful selection of confounding variables. A confounder (or common cause) is a variable that influences both the intervention and the outcome. Therefore, when adjusting for specific parameters in such an analysis, reasonable distinctions have to be made between a confounder (which needs to be adjusted for) and a mediator (which lies on the causal path between intervention and outcome) or a collider (a variable influenced by both the intervention and the outcome). Hence, if one makes adjustments based on mediators or colliders, this could introduce bias in the effect estimate [21,26]. For a better understanding of the phenomenon, Figure 1 briefly presents a graphical causal diagram differentiating between confounders, mediators, and colliders.

In the current case, based on the structure of MARK-AGE data, previous studies involving the analysis of MARK-AGE data, and domain knowledge, the chosen confounders were sex, age, BMI, WTHR, relevant plasma levels of vitamins and trace elements, and specific lifestyle factors [4,5,6,27]. Prior to the effect estimation through IPTW, a Factor Analysis of Mixed Data (FAMD) dimensionality reduction technique was applied for an optimized convergence, since both continuous and categorical variables could be found in the list of confounders [6,20]. Table 2 presents the complete list of variables based on which the effect was adjusted.

After applying the IPTW algorithm for all specified cases, the effect estimate was validated (refuted) based on the random common cause method from the DoWhy package, for which a random confounder (common cause) is added and the effect is recalculated with the updated data. Ideally, the estimated effect through random common cause should be identical (or almost the same) to the initial effect (without adding the random common cause), therefore testing the robustness of the estimated effect [21]. After applying the random common cause method, for each case, the percentual variation in the effect estimated through this refutation method was computed based on the formula presented in Equation (1) [20].

(1) $$Var(\%)=\frac{{Effect}_{initial}-{Effect}_{RCC}}{{Effect}_{initial}}\times 100$$

where *Var*(%) = percentual variation in the effect estimated through the random common cause method; *Effect_initial_* = the initial estimated effect, adjusted for the confounders specified in Table 2; and *Effect_RCC_* = the estimated effect after adding a random common cause (confounder).

## 3. Results

Table 3 presents the general characteristics of the most important demographic, biochemical, clinical, and anthropometric parameters of the study population (1326 subjects). Since all parameters followed a non-Gaussian distribution, values are expressed as median (IQR).

In the first phase, we undertook a preliminary analysis of the age- and sex-related distributions of values, measured for the main studied parameters: LDLox, NOx, % of telomeres shorter than 3 kb (CST), initial DNA integrity (%), and DNA damage after 3.8 Gy (%). The results presented in the Supplementary Material (Figures S1–S10) show a high variability in terms of the parameter values (median, first quartile, third quartile, and outliers) for each sex and each of the four analyzed age decades (35–44, 45–54, 55–64, and 65–74). For example, Figure S1 (LDLox value representation per age decade for females) showed a steady increase in the median values from 35 to 44 until the 55–64 decade, followed by a decrease within the 65–74 decade; there were also more outliers within the 35–44 and 45–54 age decades. On the other hand, Figure S2 (LDLox value representation per age decade for males) highlighted a constant decrease from the first decade (35–44) until the last decade (65–74), with more outliers within the 55–64 and 65–74 groups. The graphical representation of plasma NOx (Figures S3 and S4) showed similar patterns, with the notable exception of the male sex, for which the median values were very similar for all four decades and the outliers were also more evenly distributed, even though the interquartile range and the number of outliers for the 65–74 group were lower than for the other three age groups. On the other hand, less standard patterns were observed for the graphical representations of the three outcomes from the IPTW algorithm (Figures S5 and S6—% of telomeres shorter than 3 kb; Figures S7 and S8—initial DNA integrity; Figures S9 and S10—DNA damage after 3.8 Gy), even though there was a constant high number of outliers for all age decades for the % of telomeres shorter than 3 kb, no outliers for the initial DNA integrity, and only a few outliers for the DNA damage after 3.8 Gy.

**Figure 2:**
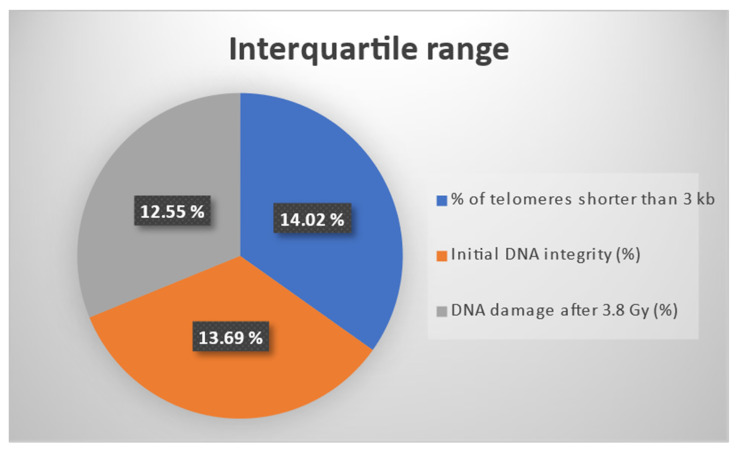
Interquartile ranges for three percentage type outcomes: % of telomeres shorter than 3 kb, initial DNA integrity, and DNA damage after 3.8 Gy.

For an enhanced visualization of the three outcomes considered for the effect estimation through the IPTW algorithm, Figure 2 presents the interquartile ranges (IQRs) for the three biomarkers: % of telomeres shorter than 3 kb, initial DNA integrity, and DNA damage after 3.8 Gy. It should be mentioned that all three markers were percentage-type data. The IQR was computed as the difference between the third and the first quartile. It should be noted that the three IQRs had similar values.

**Figure 3:**
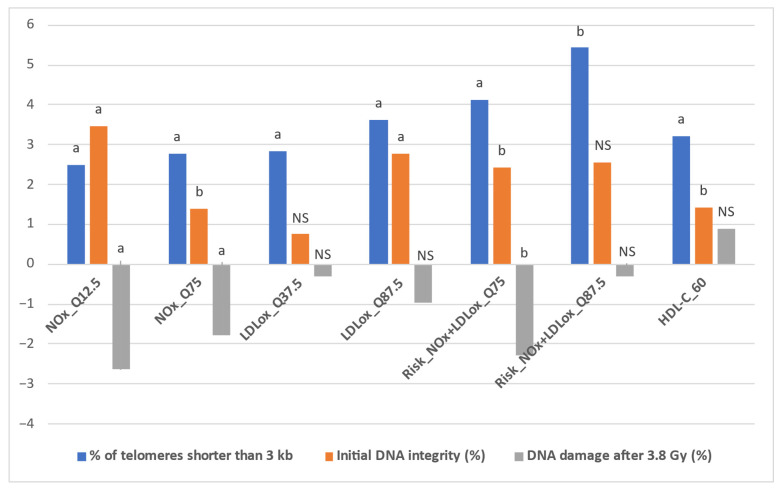
Representative situations for IPTW comparatively estimated effects. Statistical significance: ^a^ *p* < 0.01; ^b^ *p* < 0.05; NS = non-significant.

Table 4 presents the detailed results obtained after implementing the IPTW algorithm for all intervention thresholds and outcomes (estimated effect—the impact of the specific status, defined in Table 1, on each outcome, along with the *p* value for statistical significance, set at *p* < 0.05). In addition, Figure 3 depicts the representative situations for which the IPTW algorithm yielded relevant results. These results were obtained through the random common cause method, along with the percentual increase or decrease when compared to the initial effects. In this respect, Table S1 (Supplementary Material) presents the estimated effects of the intervention thresholds on the three outcomes obtained after implementing the IPTW algorithm with the addition of a random confounding variable to the initial confounder list.

First, when comparatively assessing the impact of LDLox and NOx thresholds on the % of telomeres shorter than 3 kb, NOx achieved an increase of over 2% in only two situations (2.492% for NOx_Q12.5 and 2.786% for NOx_Q75, both statistically significant, *p* < 0.01), while for LDLox a total of five such situations were identified (LDLox_Q12.5: 2.364%; LDLox_Q25: 2.45%; LDLox_Q37.5: 2.833%; LDLox_Q50: 2.705%; LDLox_Q87.5: 3.624%, all statistically significant, *p* < 0.02). However, when combining the LDLox and NOx thresholds, the increases in the % of telomeres shorter than 3 kb were more pronounced. All seven combined thresholds (based on the seven octiles: Q12.5, Q25, Q37.5, Q50, Q62.5, and Q87.5) led to a statistically significant increase of at least 2%, with a minimum of 2.335% for Risk_NOx + LDLox_Q62.5 (combined threshold—concomitant status of NOx over the Q62.5 octile of NOx and LDLox over the Q62.5 octile of LDLox), while the highest values were obtained for Risk_NOx + LDLox_Q75 (4.132%, *p* = 0.002) and Risk_NOx + LDLox_Q87.5 (5.434%, *p* = 0.03) (Table 4).

In terms of validation of the estimated effects (Table S1, Supplementary Material), robust values were obtained, with an absolute percentual variation in the effect lower than 2% in all situations where statistical significance was reached for the initial effect.

It is also relevant to display the age-related analysis of telomere shortening and DNA damage response (DDR) in association with LDLox and NOx. Table 5 highlights the relationships between the individual values of LDLox, NOx, and the three genomic instability markers, calculated per chronological age decade, in the MARK-AGE sample population (age range 35–75 years old). In addition, for a broader highlight of the associations, Table 6 shows the Spearman’s correlation coefficients between the individual values of LDLox, NOx and the three genomic instability markers on the entire cohort of 1326 individuals.

As a general feature, the correlation coefficients displayed a similar pattern to that obtained within the causality analysis. An interesting observation was that there are more statistically significant associations in the case of younger subjects (35–44 years old). Moreover, in elderly subjects (65–74 years old), only plasma NOx was significantly positively correlated with the PBMC’s DNA integrity and inversely correlated with DNA damage response after irradiation. In the age decade 65–74 years, a significant but weak association was identified between LDLox and DNA integrity. The pattern was similar on the entire RASIG cohort, with small, positive, but significant interrelations between serum LDLox and the percentage of CST, as well as between plasma NOx and initial DNA integrity, while NOx levels were inversely correlated with the DNA damage response after irradiation.

## 4. Discussion

In a previous study conducted on the MARK-AGE sample population (age range 40–75 years old), we established that LDL susceptibility to oxidation (LDLox) is a valuable potential biomarker indicating the cardiometabolic risk associated with aging. In this respect, combined ratio- and threshold-based markers between LDLox and HDL cholesterol had a higher predictive value than individual HDL cholesterol values and thresholds in several situations [5]. Therefore, in the present study we focused on researching the possible specific causal effects of LDLox and metabolization products of nitric oxide (NOx), measured in plasma samples, on well-acknowledged genomic instability markers associated with aging, as assessed in peripheral blood mononuclear cells (PBMCs).

Causal inference approaches are more complex to implement when compared to the predictive analysis of the cardiometabolic risk outcomes, as the main aim of the causal inference is to estimate, with a low risk of bias, the impact (effect) of a treatment (intervention—in our case, LDLox and NOx) on an outcome (in our case, the three genomic instability markers: the % of critically short telomeres (CST), the initial DNA integrity and the DNA damage after irradiation). Taking into account the accurate and standardized methodology based on which the Inverse Probability of Treatment Weighting (IPTW) algorithm was applied, it is reasonable to state that, for the specific LDLox and NOx thresholds for which the impact on the genomic instability markers was statistically significant, the estimated effect was robust to unobserved confounders [6,21].

Overall, in the current research, we found significant causal relationships between LDLox levels (the degree of LDL damage susceptibility to induced oxidative stress) and the % of CST (LDLox increased the % of CST for all studied LDLox levels/thresholds). Conversely, almost all NOx levels (thresholds) were found to have a protective effect on both DNA markers, increasing the initial DNA integrity and decreasing the DNA damage after irradiation.

Recent research emphasizes that age, sex, and the complex interrelations between lifestyle factors have a significant impact on telomere length and, therefore, are also able to influence the percentage of critically short telomeres [28]. Hence, the specific observed patterns resulting from the age- and sex-related value distributions of the studied biomarkers seem to validate the reasoning behind choosing specific octile-based thresholds for LDLox and NOx while maintaining continuous values for the three outcomes (% of CST, initial DNA integrity, and DNA damage after 3.8 Gy), since LDLox and NOx showed clearer increases and decreases in terms of median values and interquartile ranges, reflecting the data dispersion per age decade.

In terms of the results obtained by applying the causal inference algorithm, it should be noted that different results were obtained for LDLox and NOx when comparing their impact on the three genomic instability markers.

The lower impact of NOx on telomere shortening when compared to LDLox might be explained by the fact that nitric oxide may have a beneficial effect in specific conditions, stimulating telomerases and delaying endothelial cell senescence [29], while in other situations it might contribute to telomere shortening and DNA damage through the production of reactive oxygen species, which could also create a favorable environment for LDL oxidation [4,30,31]. Indeed, after adjusting for age and sex, the study conducted by Nawrot et al. showed that in vivo circulating oxidized LDL (oxLDL) has an inverse relationship with telomere length [3]. Nevertheless, Hong et al. reported no involvement of nitric oxide in the modulation of telomerase activity, even though the research was not performed in vivo [32]. In addition, regarding the impact of high blood pressure, low HDL cholesterol (HDL-C), high LDL cholesterol (LDL-C), and HOMA-IR, it is worth mentioning that in the current study the only threshold for which statistical significance was reached in terms of impact on telomere length was the status of HDL-C < 60 mg/dL (HDL-C_60), with a percentual increase of 3.214 (*p* = 0.001), even though the effect was smaller than the highest values obtained for the LDLox-based threshold and the combined thresholds based on NOx and LDLox octiles. Indeed, in several studies there was a positive correlation between HDL cholesterol values and leukocyte telomere length; however, they were not undertaken on European cohorts [33,34]. Nevertheless, the vast majority of the studies, whether examining the relationship between nitric oxide, oxidized LDL, or HDL cholesterol and telomerase activity or telomere length, failed to adjust for a significant number of confounders [3,33,34]. Therefore, the novelty of the present study is the fact that we managed, due to the standardized approach employed during the MARK-AGE data collection process, to implement an adjusted analysis of the interventions and outcomes based on a significant number of relevant common causes.

Secondly, the results obtained after estimating the impact of LDLox- and NOx-based thresholds on initial DNA integrity and radiation-induced DNA damage, it is worth mentioning that NOx yielded a significantly higher effect when compared to LDLox. With regard to this, the impact of NOx on initial DNA integrity yielded significant increases in almost all situations excepting the highest threshold of NOx, thus suggesting a more pronounced protective effect for moderate values of plasma NOx. On the other hand, the impact of LDL oxidation on initial DNA integrity only reached statistical significance in the case of high levels of LDLox, with the highest effect being obtained for the maximum octile. The combined thresholds of NOx and LDLox octiles reached statistically significant intermediate values between the ones obtained for LDLox and NOx, with a maximum for Risk_NOx + LDLox_Q25. The status of HDL cholesterol under 60 mg/dl and the status of LDL cholesterol over 100 mg/dL were the only traditional vascular aging markers where statistical significance was reached in terms of an impact on a significant increase in initial DNA integrity (HDL-C_60) and a significant decrease in initial DNA integrity (LDL-C_100), even though the impact was smaller than for LDLox, NOx, or the combined LDLox- and NOx-based thresholds.

Nevertheless, the analysis of the impact of NOx- and LDLox-based thresholds on the DNA damage after 3.8 Gy yielded similar effects when compared to the impact on the initial DNA integrity, suggesting a decreased susceptibility to radiation-induced DNA damage with higher serum values of NOx and LDLox, respectively. Similarly to the impact on initial DNA integrity, NOx-based thresholds reached significantly higher effects in terms of magnitude compared to LDLox-based thresholds. Meanwhile, LDLox-based thresholds yielded lower effects and no threshold based on LDLox yielded statistical significance, while the combined LDLox and NOx thresholds had a similar impact to the ones of NOx. The more pronounced effect of NOx in reducing DNA damage after 3.8 Gy could be explained by the fact that nitric oxide might increase the production of tumor suppressor protein p53, which has the ability to interrupt the cell cycle, facilitating DNA repair mechanisms [35,36]. Nevertheless, in specific conditions, high levels of nitric oxide might exert DNA damage effects through peroxynitrite (ONOO^−^), the main NO-based oxidant in the majority of situations and a very deleterious free radical. It is acknowledged that the effects of DNA damage significantly depend on the concentration of the pro-oxidant NOx metabolites, the time of the exposure, and on the potential of the cell to trigger specific defense mechanisms against the deleterious impact of nitroxidative stress [37]. Therefore, these aspects might partly explain the fact that, in the current study, plasma NOx had a protective effect on both the initial DNA integrity and after irradiating the DNA with 3.8 Gy. On the other hand, in terms of the effect of LDL oxidation on DNA integrity, a study conducted by Inoue et al. reported that in dyslipidemic subjects oxLDL exerts an immunological response to DNA damage that is independent of serum LDL-C [38], possibly explainable by the mediation of endothelial dysfunction [39].

The present causality analysis is strengthened by the proof that the age-related analysis of telomere shortening and DNA damage response (DDR) in association with LDLox and NOx confirmed the high and exclusive impact of LDLox on telomere shortening, especially in young subjects, and the high and exclusive impact of NOx on DNA integrity and damage response in elderly subjects. With regard to the impact of LDL oxidation on telomeres, the present study confirms the effects reported in previous clinical studies [3]. Experimental studies suggest that the NOx-DDR interrelationship could be mediated (modulated) by cellular signaling pathways relevant to aging, such as SIRT1. Indeed, SIRT1 enhances endothelial nitric oxide synthase (eNOS) expression and NO bioavailability, but NO may also directly modulate SIRT1 expression [40]. To the same extent, it is acknowledged that SRT1 plays a crucial role in the DDR by promoting DNA repair [41].

The main biological outcome of the present study is the clear indication of the impact of LDL oxidation and NO metabolism on genomic instability markers in human subjects, evidenced within a large-scale population study. The novelty is that both parameters (evaluated at the systemic level), namely LDLox and NOx as key players involved in endothelial dysfunction and atherogenesis, were analyzed to explore their influence on cellular DNA. It is acknowledged that, from a physiopathological viewpoint, LDL oxidation and NO could have antagonistic actions within the vascular microenvironment [4]. In this sense, the present study demonstrated that LDLox and NOx also displayed distinct effects on specific genomic instability markers such as telomere length and DNA integrity. Although these DNA-based markers were evaluated in isolated PBMCs, we could extrapolate this situation to the adjacent endothelial cells. Therefore, this approach has special biological relevance by proving that LDLox and NOx exert a significant role on genome integrity, which is of critical importance in vascular cell function/senescence.

Hence, taking into account the results obtained by implementing the IPTW algorithm, one of the main findings of the current study is that the use of a wide range of thresholds could indicate that even small changes in LDLox and NOx values could have a meaningful effect on telomere length and DNA integrity. To our knowledge, this is the first study conducted on a European population that estimates the impact of specific oxidative stress parameters on genomic instability markers by adjusting for a relevant number of blood serum assays and sociodemographic and lifestyle factors. It should be noted that, due to the standardized approach during the MARK-AGE data collection process, we implemented an adjusted analysis based on a significant number of relevant common causes of the interventions and outcomes.

An important strength of the current research is the approach taken to vascular dysfunction/aging by considering multiple DNA-based markers as outcomes within the same analysis. Another element of originality is the fact that, within the MARK-AGE cohort, an in vitro method was employed for the evaluation of the susceptibility to oxidation of LDL (LDLox), which is a biochemical parameter that could more accurately indicate its intrinsic atherogenic properties, as compared to the circulating oxidized LDL (oxLDL) usually measured in vivo [5].

Nevertheless, given the main limitations of the analysis (the relatively small number of subjects, under 1500, despite being comparable to or higher than those in other similar studies [3,33,34,38], the presence of relevant unobserved confounders cannot be completely ruled out [6,21]), future studies must focus on designing randomized studies and enlarging the cohort size, consequently aiming to validate clinically relevant threshold combinations for LDLox and NOx, which could optimally explain vascular dysfunction/aging and other health issues related to telomere shortening and DNA damage.

## 5. Conclusions

In the present study, we managed to show the specific causal relationships between both the susceptibility to oxidation of LDL and the metabolization products of NO and genomic instability markers, defined as the fraction of critically short telomeres, DNA integrity in normal conditions, and radiation-induced DNA damage, through the use of a method with a low risk of bias on a representative MARK-AGE cohort from the general population. While LDLox showed significant harmful effects on telomeres, the analysis demonstrated protective effects of NOx in terms of native DNA integrity, with an additional reduction in DNA damage after applying a radiation level of 3.8 Gy. All effects were evidenced within a stratified analysis and were higher on average than the effects of traditional cardiometabolic risk/vascular aging markers/determinants such as high blood pressure, high insulin resistance index (HOMA-IR), high LDL cholesterol, and low HDL cholesterol. Therefore, the current study has the potential to open up future research aiming to define and validate the specific risk values of such non-traditional biomarkers and translate their complex role within the intricate mechanisms of vascular aging into a biomedical application.

## Figures and Tables

**Table 1:** Thresholds for each intervention considered in IPTW algorithm.

Intervention Threshold Name	Sign	Intervention Threshold Value	% of Subjects
NOx_Q12.5	≥	14.885 μmol/L	87.41
NOx_Q25	≥	18.875 μmol/L	74.81
NOx_Q37.5	≥	22.295 μmol/L	62.22
NOx_Q50	≥	25.92 μmol/L	49.70
NOx_Q62.5	≥	29.72 μmol/L	37.25
NOx_Q75	≥	34.35 μmol/L	24.89
NOx_Q87.5	≥	41.845 μmol/L	12.29
LDLox_Q12.5	≥	11.66 nmol MDA/mL	87.56
LDLox_Q25	≥	13.705 nmol MDA/mL	75.26
LDLox_Q37.5	≥	15.445 nmol MDA/mL	62.75
LDLox_Q50	≥	17.59 nmol MDA/mL	50.00
LDLox_Q62.5	≥	20.15 nmol MDA/mL	37.41
LDLox_Q75	≥	23.24 nmol MDA/mL	24.89
LDLox_Q87.5	≥	27.615 nmol MDA/mL	12.59
Risk_NOx + LDLox_Q12.5	≥	-	76.32
Risk_NOx + LDLox_Q25	≥	-	56.33
Risk_NOx + LDLox_Q37.5	≥	-	40.27
Risk_NOx + LDLox_Q50	≥	-	26.17
Risk_NOx + LDLox_Q62.5	≥	-	15.76
Risk_NOx + LDLox_Q75	≥	-	7.69
Risk_NOx + LDLox_Q87.5	≥	-	1.73
HBP_systolic_stage1	≥	140 mmHg	37.10
HBP_diastolic_stage1	≥	90 mmHg	23.30
HBP_uncontrolled_stage1	≥	Subjects with uncontrolled BP (at least stage 1)	41.93
HBP_stage1	≥	Subjects with both controlled and uncontrolled BP (at least stage 1)	49.25
HDL-C_40_50	<	40 mg/dL (males) OR 50 mg/dL (females)	3.39
HDL-C_60	<	60 mg/dL	28.81
LDL-C_70	≥	70 mg/dL	97.06
LDL-C_100	≥	100 mg/dL	81.30
LDL-C_116	≥	116 mg/dL	64.78
LDL-C_190	≥	190 mg/dL	2.19
HOMA_1.9	≥	1.9	19.98
HOMA_2.9	≥	2.9	8.90

Legend: Q, quantile; NOx, nitric oxide metabolic pathway products; LDLox, low-density lipoprotein susceptibility to oxidation; Risk_NOx + LDLox, combined thresholds between octiles for serum LDLox and NOx—concomitant status of NOx and LDLox above (or at least equal to) mentioned values; HBP, high blood pressure; HDL-C, HDL cholesterol; LDL-C, LDL cholesterol; HOMA, Homeostasis Model Assessment—Insulin Resistance; MDA, malondialdehyde; Sign, way in which the threshold was considered for the intervention effect estimate and frequency distribution (HDL cholesterol was the only variable for which the intervention was set based upon values lower than the specific threshold).

**Table 2:** Complete list of confounding variables for IPTW algorithm.

Confounder
Sex
Chronological age
BMI
WTHR
Alpha-carotene
Beta-carotene
Gamma-tocopherol
Alpha-tocopherol
Lycopene
Retinol
25-Hydroxy-Vitamin-D
Fe
Se
Cu/Zn ratio
Lifestyle factors/characteristics: current smoker (yes/no), previous smoker (yes/no), smoking years, consume_bread_brown, consume_bread_white, consume_bread_whole, consume_cake_pie, consume_candies_sweets, consume_cheese, consume_dairy_products, consume_eggs, consume_fish, consume_fries_fried_potatos, consume_fruit, consume_icecream_dessert, consume_meat, consume_other_supplements, consume_salty_snacks, consume_sausages, consume_vegetables, consume_vitamin_supplements, consume_white_rice, drink_beer_never, drink_cola_soft_never, drink_never_juice, drink_never_water, drink_other_alco_never, drink_wine_never, day_activities_bathing_dressing_self, day_activities_bending_kneeling, day_activities_lifting_groceries, day_activities_moderate, day_activities_one_stairs, day_activities_several_stairs, day_activities_vigorous, day_activities_walking_half_mile, day_activities_walking_hundred_yards, day_activities_walking_several_miles, lives_with_children, lives_with_friends, lives_with_relatives, lives_with_spouse, marital_status_never, marital_status_divorced, marital_status_widow, marital_status_married, housing_apartment, housing_house, housing_special, ip_education_Never, ip_education_university degree, ip_education_Finished elementary, ip_education_First stage, ip_education_Second stage, feel_calm_peaceful, feel_full_of_life, feel_happy_person

BMI—body mass index; WTHR—waist-to-hip ratio; lifestyle factors beginning with “consume”—quantitative consumption of the specific food (frequency per month was converted to continuous data depending on the number of times per month the specific food was consumed: daily consumption: 30; 4–6 times per week: 20; 1–3 times per week: 8; 1–3 times per month: 2; never: 0); lifestyle factors beginning with “drink_never”—whether subject never drinks the specific beverage (categorical variable—yes/no); lifestyle factors beginning with “day_activities”—how limited subject was with regard to the specific physical activity (ordinal variable—categories: not limited, little limited, limited at a high degree); lifestyle factors beginning with “lives_with”—whether subject lives with the specific category (spouse, children, other relative, friends) (categorical variable—yes/no); lifestyle factors beginning with “marital_status”—marital status of subject (categorical variable—yes/no); lifestyle factors beginning with “housing”—which type of building subject lives in (categorical variable—yes/no); lifestyle factors beginning with “ip_education”—whether subject has specific degree (categorical variable—yes/no); lifestyle factors beginning with “feel”— self-reported level of happiness (ordinal variable—categories: none time, little time, some time, good bit time, most time, all time).

**Table 3:** General characteristics of study population in terms of most relevant demographic, biochemical, and clinical parameters.

Parameter	Median (IQR)
Chronological age, years	55.563 (18.566)
LDLox, nmol MDA/mL	17.565 (9.46)
NOx (NO_2_^−^ + NO_3_^−^), μmol/L	25.808 (15.505)
CST, %	19.733 (14.017)
Initial DNA integrity, %	75.874 (13.69)
DNA damage after 3.8 Gy, %	41.224 (12.551)
HDL cholesterol, mg/dL	70.49 (25.26)
LDL cholesterol, mg/dL	126.864 (41.151)
HOMA-IR	1.121 (0.953)
Systolic BP, mmHg	130 (26)
Diastolic BP, mmHg	80 (15)
BMI, kg/m^2^	25.352 (5.371)
WTHR	0.906 (0.105)

BMI—body mass index; WTHR—waist-to-hip ratio; CST—% of telomeres shorter than 3 kb; BP—blood pressure; HOMA-IR—Homeostasis Model Assessment—Insulin Resistance.

**Table 4:** Estimated effects of intervention thresholds on three outcomes, obtained after implementing IPTW algorithm.

Intervention Threshold Name	% of Telomeres Shorter than 3 kb	Initial DNA Integrity (%)	DNA Damage After 3.8 Gy (%)
**NOx_Q12.5**	**2.492 (*p* = 0.008)**	**3.478 (*p* = 0.001)**	**−2.64 (*p* = 0.002)**
NOx_Q25	1.429 (*p* = 0.045)	**3.608 (*p* = 0.001)**	**−1.803 (*p* = 0.002)**
NOx_Q37.5	1.064 (*p* = 0.086; NS *)	**3.326 (*p* = 0.001)**	**−1.646 (*p* = 0.001)**
**NOx_Q50**	**1.619 (*p* = 0.019)**	**2.313 (*p* = 0.001)**	**−1.412 (*p* = 0.002)**
**NOx_Q62.5**	**1.916 (*p* = 0.006)**	**1.809 (*p* = 0.001)**	**−1.83 (*p* = 0.001)**
**NOx_Q75**	**2.786 (*p* = 0.001)**	**1.391 (*p* = 0.018)**	**−1.778 (*p* = 0.003)**
NOx_Q87.5	1.68 (*p* = 0.062; NS)	0.215 (*p* = 0.406; NS)	−1.038 (*p* = 0.139; NS)
LDLox_Q12.5	**2.364 (*p* = 0.015)**	−0.632 (*p* = 0.244; NS)	1.495 (*p* = 0.051; NS)
LDLox_Q25	**2.45 (*p* = 0.002)**	1.069 (*p* = 0.055; NS)	0.145 (*p* = 0.429; NS)
LDLox_Q37.5	**2.833 (*p* = 0.001)**	0.754 (*p* = 0.099; NS)	−0.317 (*p* = 0.301; NS)
LDLox_Q50	**2.705 (*p* = 0.001)**	**1.065 (*p* = 0.027)**	−0.689 (*p* = 0.109; NS)
LDLox_Q62.5	**1.556 (*p* = 0.021)**	**1.249 (*p* = 0.013)**	−0.856 (*p* = 0.077; NS)
LDLox_Q75	**1.618 (*p* = 0.039)**	0.957 (*p* = 0.064; NS)	−0.676 (*p* = 0.154; NS)
LDLox_Q87.5	**3.624 (*p* = 0.002)**	**2.778 (*p* = 0.001)**	−0.975 (*p* = 0.135; NS)
Risk_NOx + LDLox_Q12.5	**2.726 (*p* = 0.001)**	**1.657 (*p* = 0.004)**	−0.96 (*p* = 0.087; NS)
**Risk_NOx + LDLox_Q25**	**2.373 (*p* = 0.001)**	**2.916 (*p* = 0.001)**	**−1.268 (*p* = 0.012)**
**Risk_NOx + LDLox_Q37.5**	**3.205 (*p* = 0.001)**	**2.542 (*p* = 0.001)**	**−1.074 (*p* = 0.035)**
**Risk_NOx + LDLox_Q50**	**3.481 (*p* = 0.001)**	**1.669 (*p* = 0.003)**	**−1.245 (*p* = 0.031)**
**Risk_NOx + LDLox_Q62.5**	**2.335 (*p* = 0.019)**	**1.536 (*p* = 0.026)**	**−2.17 (*p* = 0.003)**
**Risk_NOx + LDLox_Q75**	**4.132 (*p* = 0.002)**	**2.431 (*p* = 0.013)**	**−2.275 (*p* = 0.013)**
Risk_NOx + LDLox_Q87.5	**5.434 (*p* = 0.03)**	2.566 (*p* = 0.124; NS)	−0.296 (*p* = 0.448; NS)
HBP_systolic_stage1	0.003 (*p* = 0.476; NS)	0.276 (*p* = 0.337; NS)	−0.787 (*p* = 0.114; NS)
HBP_diastolic_stage1	−0.511 (*p* = 0.321; NS)	0.837 (*p* = 0.11; NS)	−0.276 (*p* = 0.342; NS)
HBP_uncontrolled_stage1	−0.338 (*p* = 0.355; NS)	0.44 (*p* = 0.214; NS)	−0.231 (*p* = 0.352; NS)
HBP_stage1	−0.011 (*p* = 0.497; NS)	0.589 (*p* = 0.152; NS)	−0.752 (*p* = 0.112; NS)
HDL-C_40_50	1.487 (*p* = 0.218; NS)	0.602 (*p* = 0.361; NS)	−2.102 (*p* = 0.1; NS)
**HDL-C_60**	**3.214 (*p* = 0.001)**	**1.419 (*p* = 0.02)**	0.883 (*p* = 0.103; NS)
LDL-C_70	−1.153 (*p* = 0.303; NS)	−1.995 (*p* = 0.117; NS)	0.191 (*p* = 0.461; NS)
LDL-C_100	−0.929 (*p* = 0.169; NS)	**−1.869 (*p* = 0.006)**	−0.373 (*p* = 0.278; NS)
LDL-C_116	−0.679 (*p* = 0.185; NS)	−1.001 (*p* = 0.052; NS)	−0.194 (*p* = 0.339; NS)
LDL-C_190	−0.218 (*p* = 0.472; NS)	0.662 (*p* = 0.367; NS)	−2.634 (*p* = 0.089; NS)
HOMA_1.9	0.549 (*p* = 0.293; NS)	1.259 (*p* = 0.055; NS)	−0.939 (*p* = 0.095; NS)
HOMA_2.9	0.312 (*p* = 0.427; NS)	0.71 (*p* = 0.248; NS)	−0.251 (*p* = 0.395; NS)

* NS = non-significant. The most important statistically significant effects are marked with bold.

**Table 5:** Spearman’s correlation coefficients (*rho*) between continuous values of LDLox, NOx, and three genomic instability markers, computed per chronological age decade, in RASIG participants.

	% of Telomeres Shorter than 3 kb	Initial DNA Integrity (%)	DNA Damage After 3.8 Gy (%)
**Age decade: 35–44 years (n = 295)** **Age median (IQR): 40.053 (4.524)**			
**LDLox, nmol MDA/mL**	**0.148 (*p* = 0.011)**	−0.04 (*p* = 0.493; NS *)	−0.041 (*p* = 0.479; NS)
**NOx, μmol/L**	−0.001 (*p* = 0.991; NS)	**0.163 (*p* = 0.005)**	**−0.121 (*p* = 0.038)**
**Age decade: 45–54 years (n = 350)** **Age median (IQR): 50.163 (4.215)**			
**LDLox, nmol MDA/mL**	**0.121 (*p* = 0.023)**	0.041 (*p* = 0.44; NS)	0.04 (*p* = 0.451; NS)
**NOx, μmol/L**	0.046 (*p* = 0.39; NS)	**0.177 (*p* = 0.001)**	−0.085 (*p* = 0.113; NS)
**Age decade: 55–64 years (n = 353)** **Age median (IQR): 60.187 (5.169)**			
**LDLox, nmol MDA/mL**	0.077 (*p* = 0.147; NS)	**0.108 (*p* = 0.043)**	−0.024 (*p* = 0.647; NS)
**NOx, μmol/L**	0.094 (*p* = 0.077; NS)	0.07 (*p* = 0.19; NS)	−0.087 (*p* = 0.102; NS)
**Age decade: 65–74 years (n = 328)** **Age median (IQR): 70.062 (5.238)**			
**LDLox, nmol MDA/mL**	0.067 (*p* = 0.226; NS)	0.077 (*p* = 0.162; NS)	−0.004 (*p* = 0.94; NS)
**NOx, μmol/L**	−0.038 (*p* = 0.488; NS)	**0.208 (*p* < 0.001)**	**−0.154 (*p* = 0.005)**

n—number of subjects; NS—non-significant; IQR—interquartile range. LDLox—low-density lipoprotein susceptibility to oxidation; NOx—nitric oxide metabolic pathway products. * NS = non-significant. The statistically significant results are marked with bold.

**Table 6:** Spearman’s correlation coefficients (*rho*) between continuous values of LDLox, NOx, and three genomic instability markers, computed on entire cohort of 1326 subjects, in RASIG participants.

	% of Telomeres Shorter than 3 kb	Initial DNA Integrity (%)	DNA Damage After 3.8 Gy (%)
**LDLox, nmol MDA/mL**	**0.109 (*p* < 0.001)**	0.053 (*p* = 0.054; NS)	0 (*p* = 0.986; NS)
**NOx, μmol/L**	0.033 (*p* = 0.224; NS)	**0.152 (*p* < 0.001)**	**−0.103 (*p* < 0.001)**

LDLox—low-density lipoprotein susceptibility to oxidation; NOx—nitric oxide metabolic pathway products. The statistically significant results are marked with bold.

## Data Availability

The data presented in this study are available on request.

## Supplementary Materials

The following supporting information can be downloaded at: https://www.mdpi.com/article/10.3390/antiox14080933/s1.

## Abbreviations

The following abbreviations are used in this manuscript:

CST	Critically short telomeres
