# Supplementary material for: Causal Inference Approaches Reveal Associations Between LDL Oxidation, NO Metabolism, Telomere Length and DNA Integrity Within the MARK-AGE Study
Source: Antioxidants (Basel). 2025 Jul 30;14(8):933. doi: 10.3390/antiox14080933 (PMC12383079; doi:10.3390/antiox14080933)
Supplement: Supplementary file 1 [file antioxidants-14-00933-s001.zip › antioxidants-3649533-supplementary.pdf]

Supplementary Material

Table S1. The estimated effects* of the intervention thresholds on the three outcomes (and the percentual effect increase or decrease when compared to the initial effects), obtained after implementing the IPTW algorithm with the addition on a random confounding variable to the initial confounders list

Intervention threshold name	% of telomeres shorter than 3 kb	Initial DNA integrity (%)	DNA integrity damage after 3.8 Gy (%)
NOx_Q12.5	**2.481 (0.441%)**	**3.479 (-0.029%)**	**-2.617 (0.871%)**
NOx_Q25	**1.425 (0.28%)**	**3.613 (-0.139%)**	**-1.781 (1.22%)**
NOx_Q37.5	**1.053 (1.034%)**	**3.345 (-0.571%)**	**-1.621 (1.519%)**
NOx_Q50	**1.619 (0.0%)**	**2.317 (-0.173%)**	**-1.411 (0.071%)**
NOx_Q62.5	**1.922 (-0.313%)**	**1.814 (-0.276%)**	**-1.833 (-0.164%)**
NOx_Q75	**2.791 (-0.179%)**	**1.396 (-0.359%)**	**-1.781 (-0.169%)**
NOx_Q87.5	**1.679 (0.06%)**	**0.215 (0.0%)**	**-1.039 (-0.096%)**
LDLox_Q12.5	2.259 (4.442%)	-0.416 (34.177%)	1.437 (3.88%)
LDLox_Q25	**2.444 (0.245%)**	1.148 (-7.39%)	0.151 (-4.138%)
LDLox_Q37.5	**2.847 (-0.494%)**	0.783 (-3.846%)	**-0.314 (0.946%)**
LDLox_Q50	**2.702 (0.111%)**	**1.064 (0.094%)**	**-0.689 (-0.0%)**
LDLox_Q62.5	**1.543 (0.835%)**	**1.261 (-0.961%)**	**-0.863 (-0.818%)**
LDLox_Q75	1.558 (3.708%)	0.983 (-2.717%)	**-0.67 (0.888%)**
LDLox_Q87.5	3.777 (-4.222%)	2.668 (3.96%)	**-0.972 (0.308%)**
Risk_NOx+LDLox_Q12.5	2.669 (2.091%)	1.722 (-3.923%)	**-0.953 (0.729%)**
Risk_NOx+LDLox_Q25	**2.358 (0.632%)**	**2.959 (-1.475%)**	**-1.255 (1.025%)**
Risk_NOx+LDLox_Q37.5	**3.197 (0.25%)**	**2.558 (-0.629%)**	**-1.083 (-0.838%)**
Risk_NOx+LDLox_Q50	**3.49 (-0.259%)**	**1.665 (0.24%)**	**-1.233 (0.964%)**
Risk_NOx+LDLox_Q62.5	**2.325 (0.428%)**	**1.545 (-0.586%)**	**-2.19 (-0.922%)**
Risk_NOx+LDLox_Q75	**4.128 (0.097%)**	**2.443 (-0.494%)**	**-2.265 (0.44%)**
Risk_NOx+LDLox_Q87.5	**5.33 (1.914%)**	**2.556 (0.39%)**	-0.273 (7.77%)
HBP_systolic_stage1	0.001 (66.667%)	**0.275 (0.362%)**	**-0.79 (-0.381%)**
HBP_diastolic_stage1	**-0.504 (1.37%)**	**0.843 (-0.717%)**	-0.268 (2.899%)
HBP_uncontrolled_stage1	**-0.338 (-0.0%)**	**0.441 (-0.227%)**	**-0.234 (-1.299%)**

HBP_stage1	-0.012 (-9.091%)	**0.589 (0.0%)**	**-0.751 (0.133%)**
HDL-C_40_50	1.453 (2.286%)	0.664 (-10.299%)	-2.221 (-5.661%)
HDL-C_60	**3.207 (0.218%)**	**1.42 (-0.07%)**	0.858 (2.831%)
LDL-C_70	-1.19 (-3.209%)	-2.022 (-1.353%)	0.09 (52.88%)
**LDL-C_100**	**-0.93 (-0.108%)**	**-1.869 (-0.0%)**	**-0.374 (-0.268%)**
LDL-C_116	-0.697 (-2.651%)	-1.026 (-2.498%)	**-0.194 (-0.0%)**
LDL-C_190	-0.203 (6.881%)	**0.656 (0.906%)**	**-2.62 (0.532%)**
**HOMA_1.9**	**0.549 (0.0%)**	**1.259 (0.0%)**	**-0.939 (-0.0%)**
HOMA_2.9	0.395 (-26.603%)	0.708 (0.282%)	-0.198 (21.116%)

* Since the random common cause method checks the sensitivity of the effect to a random unobserved confounder (while comparing the new effect to the initial effect), no p value was generated.

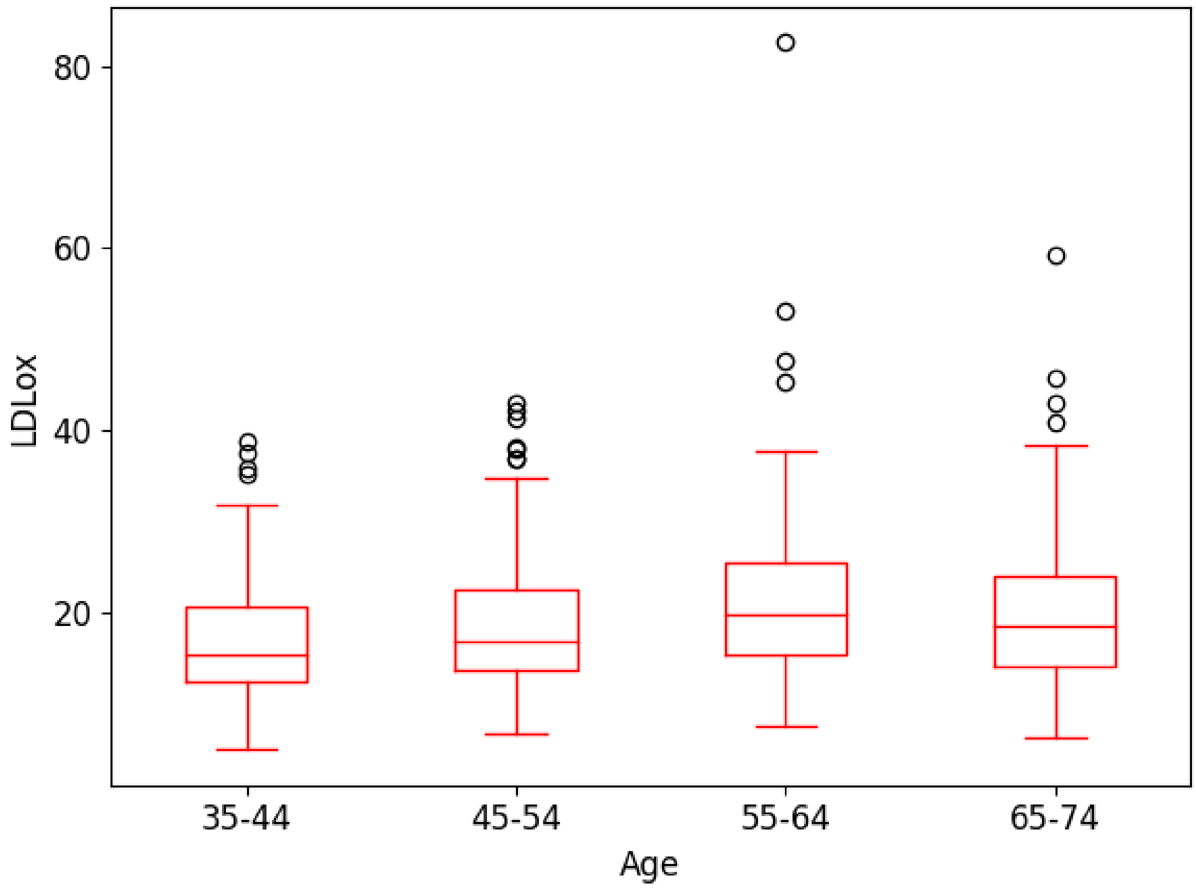

Figure S1. LDLox values representation per age decades for females (691 subjects). The boxes show the median values, the 25th and 75th percentile. Whiskers indicate the 5th and 95th percentile. Outliers are displayed for each age-group.

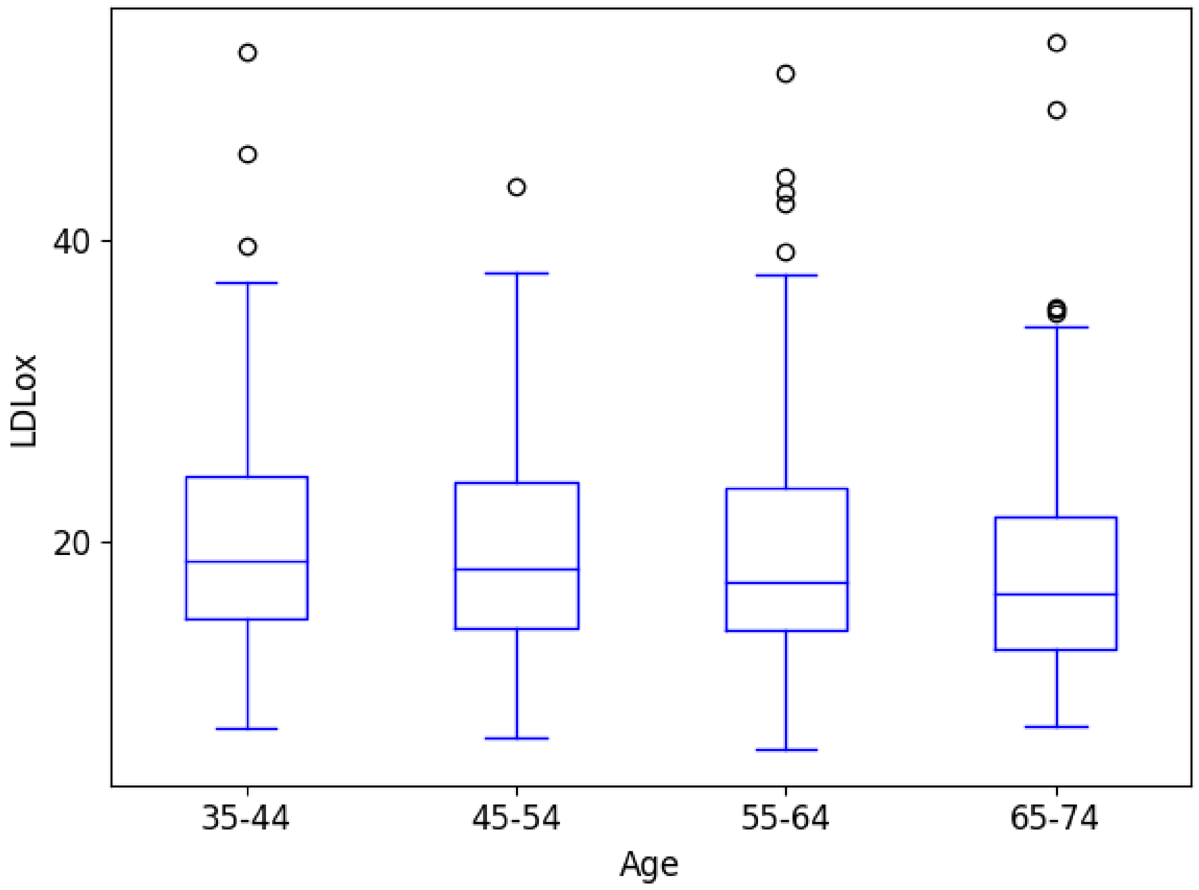

Figure S2. LDLox values representation per age decades for males (635 subjects). The boxes show the median values, the 25th and 75th percentile. Whiskers indicate the 5th and 95th percentile. Outliers are displayed for each age-group.

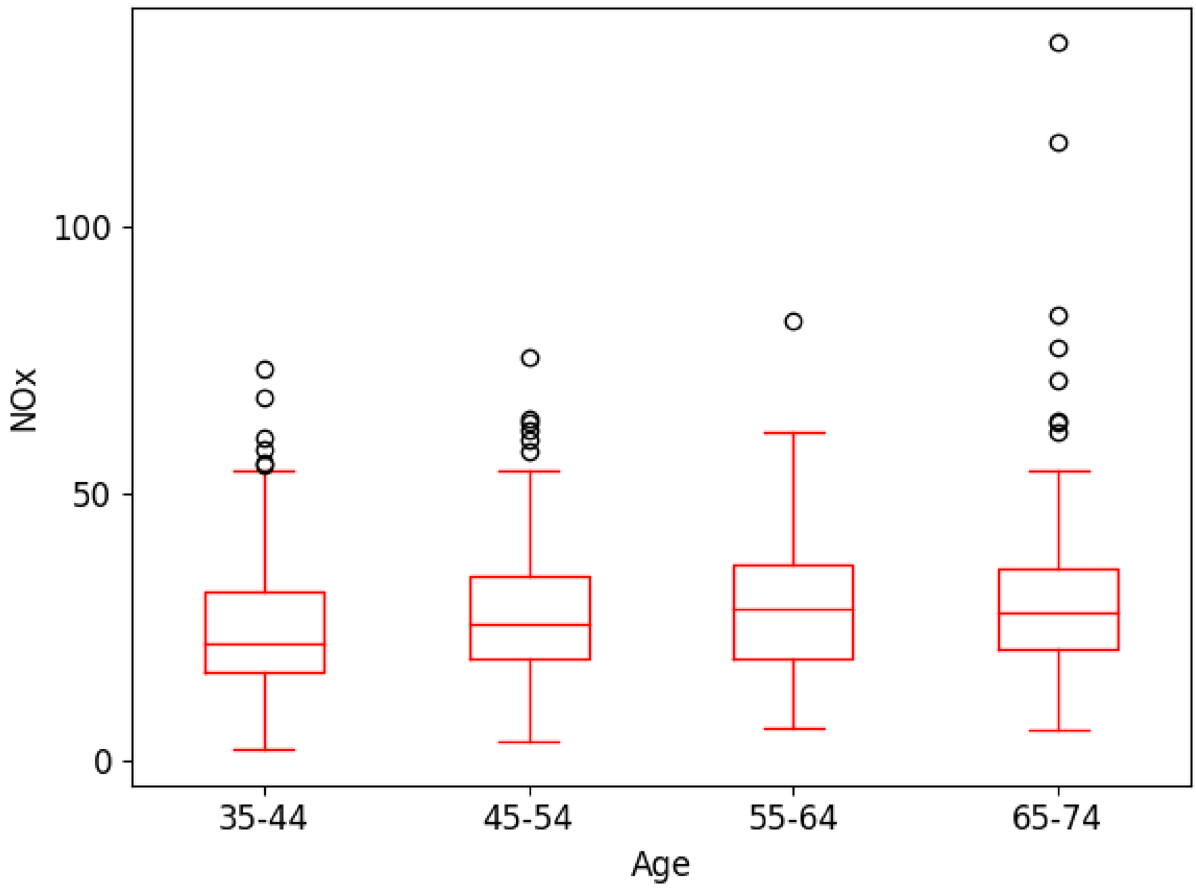

Figure S3. NOx values representation per age decades for females (691 subjects). The boxes show the median values, the 25th and 75th percentile. Whiskers indicate the 5th and 95th percentile. Outliers are displayed for each age-group.

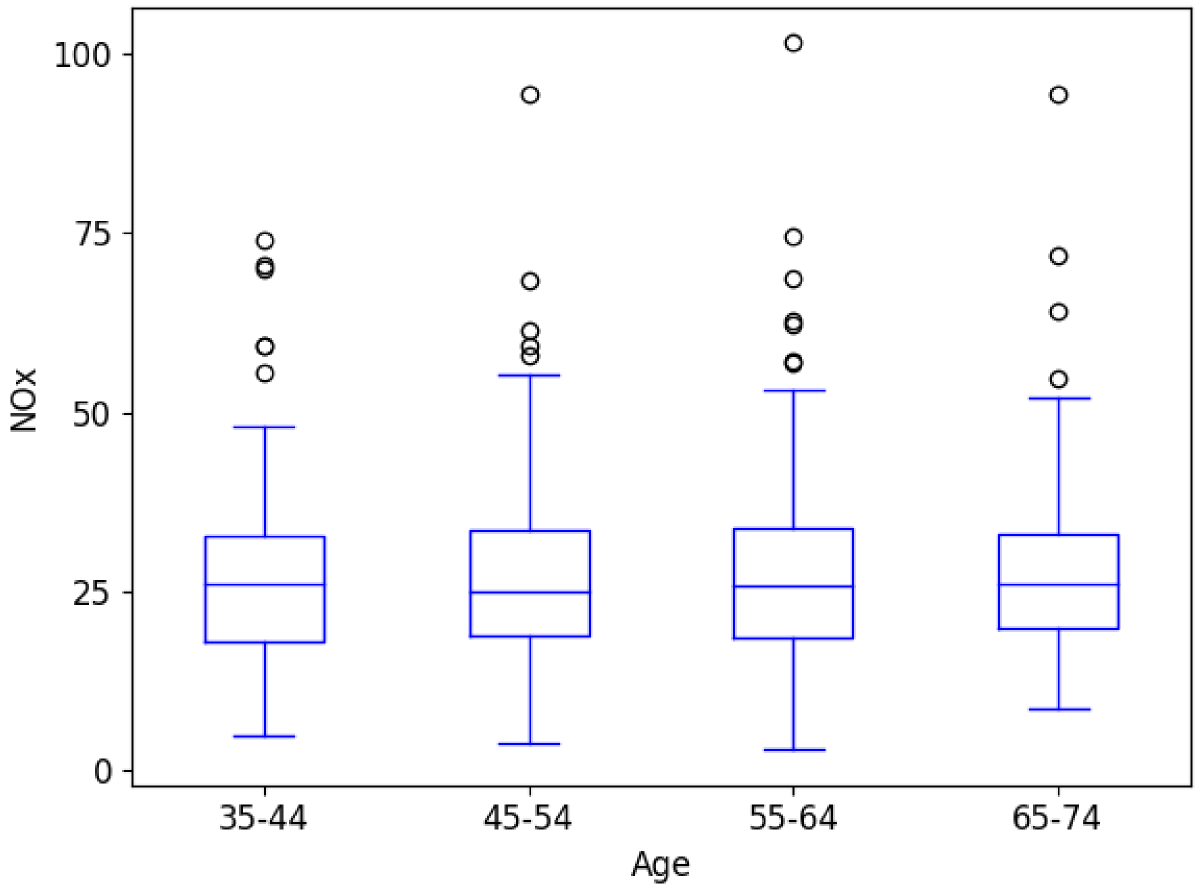

Figure S4. NOx values representation per age decades for males (635 subjects). The boxes show the median values, the 25th and 75th percentile. Whiskers indicate the 5th and 95th percentile. Outliers are displayed for each age-group.

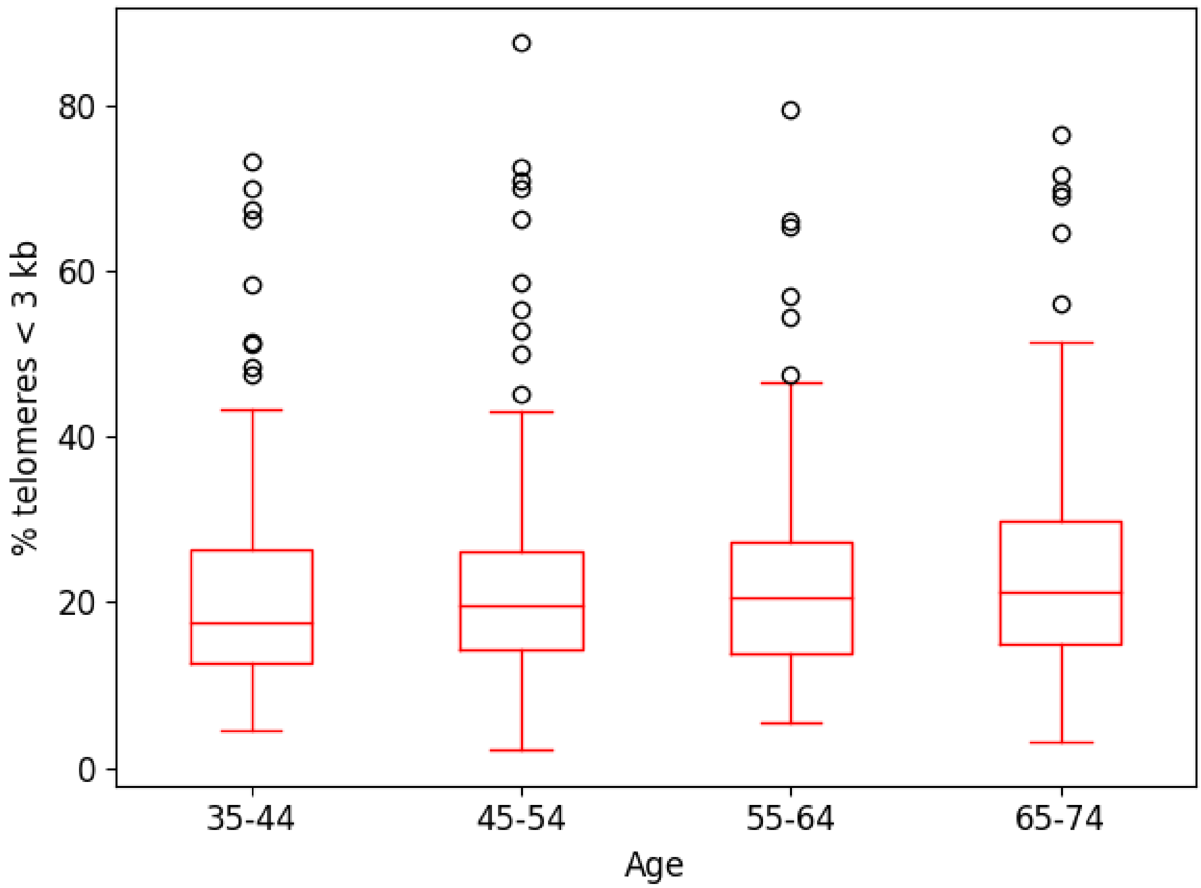

Figure S5. % of telomeres shorter than 3 kb representation per age decades for females (691 subjects). The boxes show the median values, the 25th and 75th percentile. Whiskers indicate the 5th and 95th percentile. Outliers are displayed for each age-group.

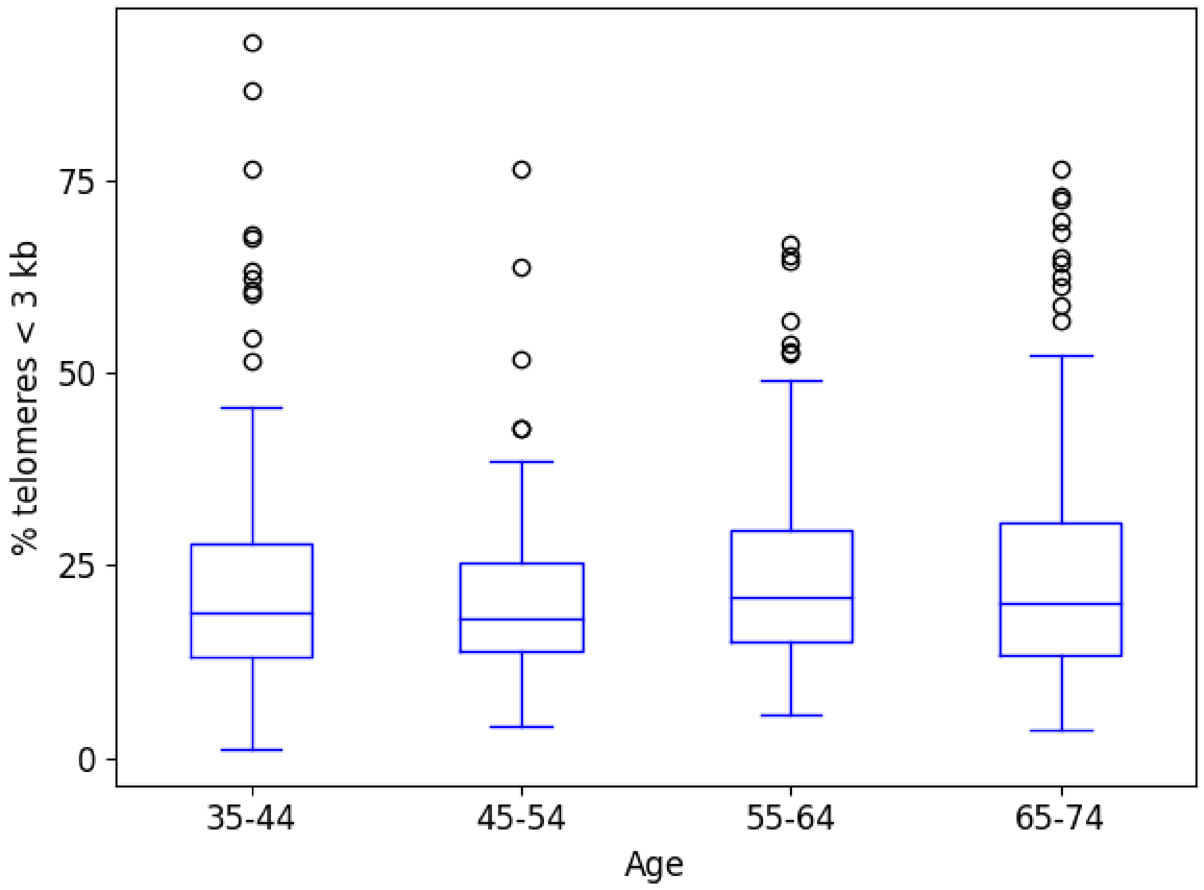

Figure S6. % of telomeres shorter than 3 kb representation per age decades for males (635 subjects). The boxes show the median values, the 25th and 75th percentile. Whiskers indicate the 5th and 95th percentile. Outliers are displayed for each age-group.

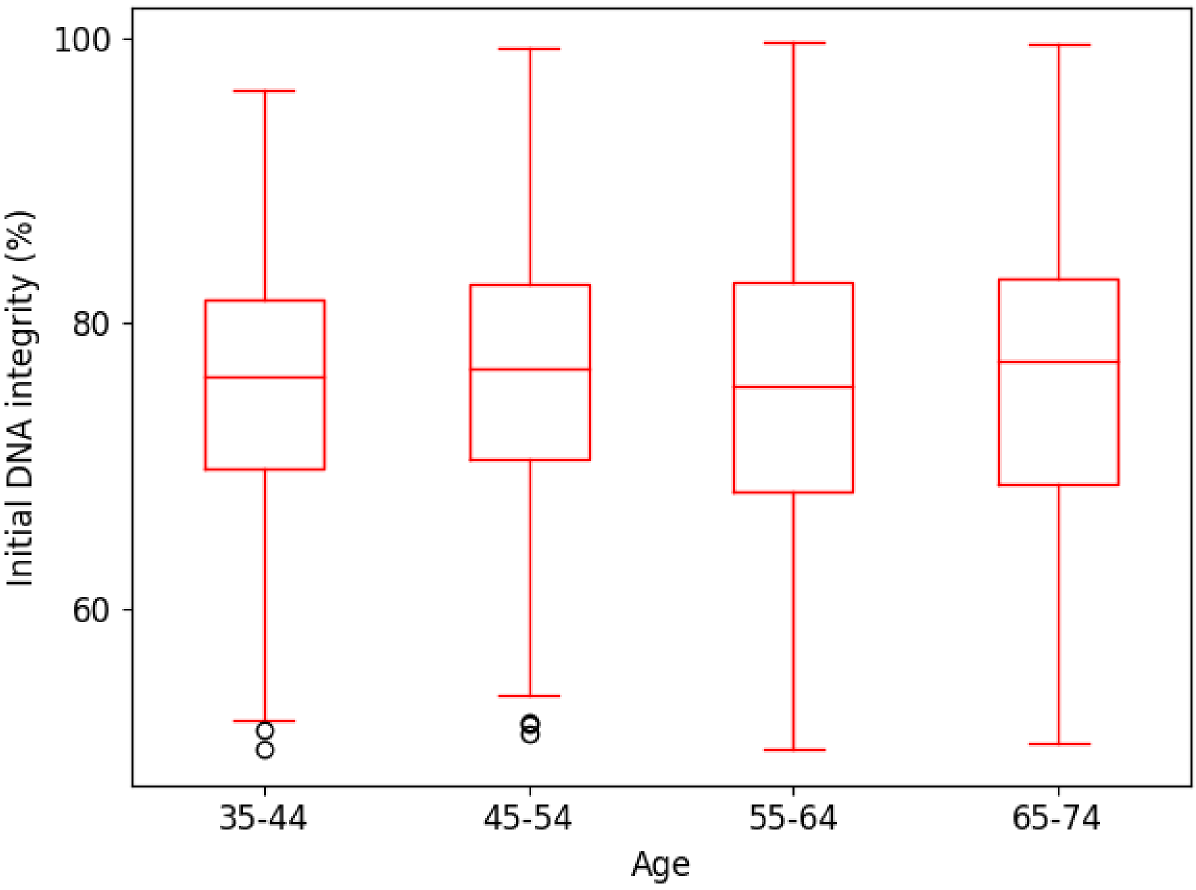

Figure S7. Initial DNA integrity (%) values representation representation per age decades for females (691 subjects). The boxes show the median values, the 25th and 75th percentile. Whiskers indicate the 5th and 95th percentile. Outliers are displayed for each age-group.

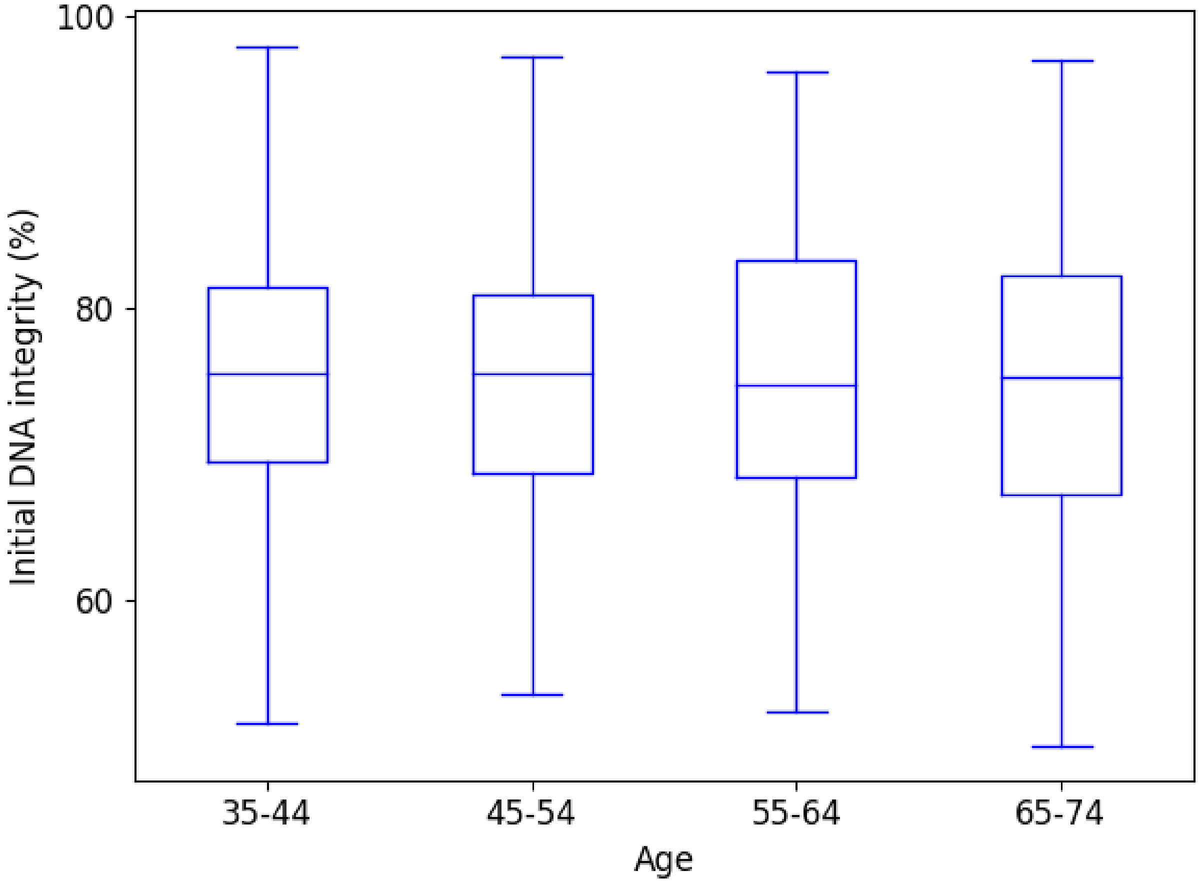

Figure S8. Initial DNA integrity (%) values representation representation per age decades for males (635 subjects). The boxes show the median values, the 25th and 75th percentile. Whiskers indicate the 5th and 95th percentile. Outliers are displayed for each age-group.

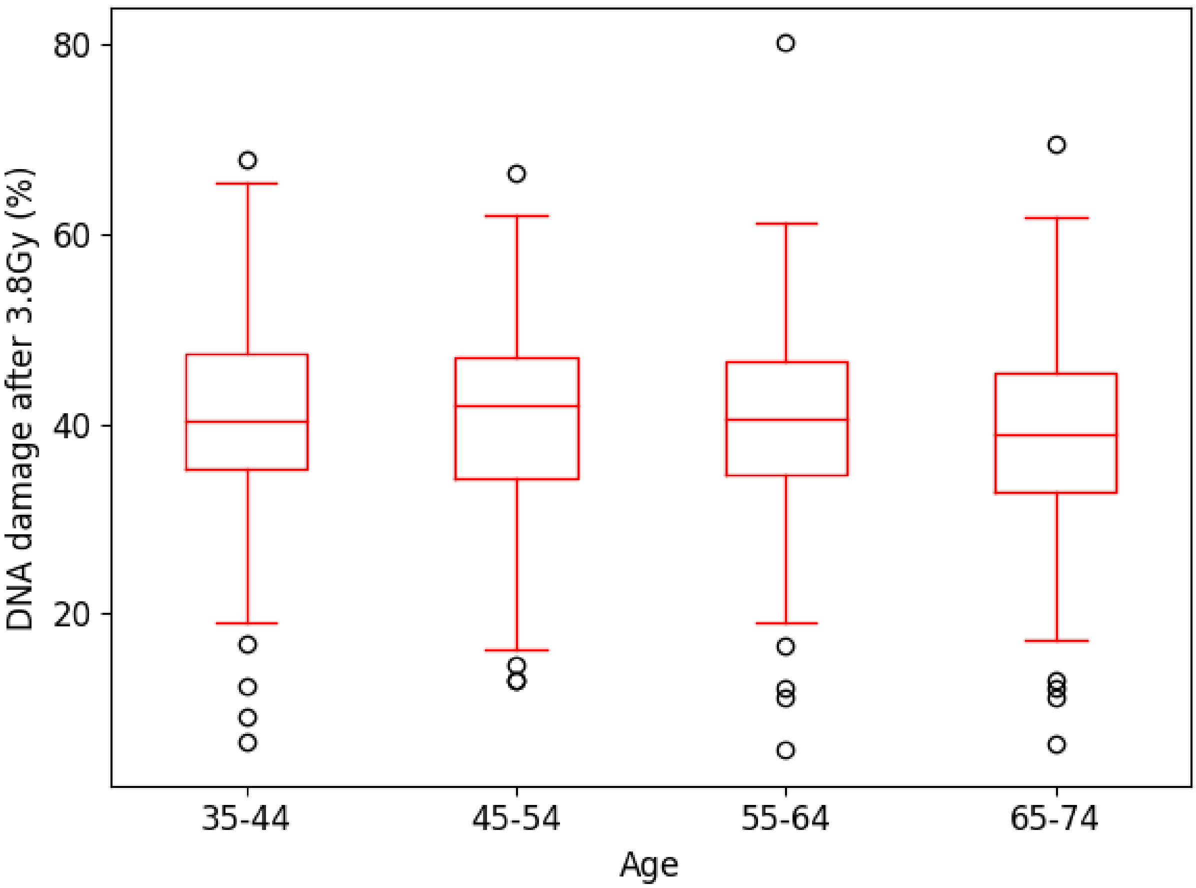

Figure S9. DNA damage after 3.8 Gy (%) values representation representation per age decades for females (691 subjects). The boxes show the median values, the 25th and 75th percentile. Whiskers indicate the 5th and 95th percentile. Outliers are displayed for each age-group.

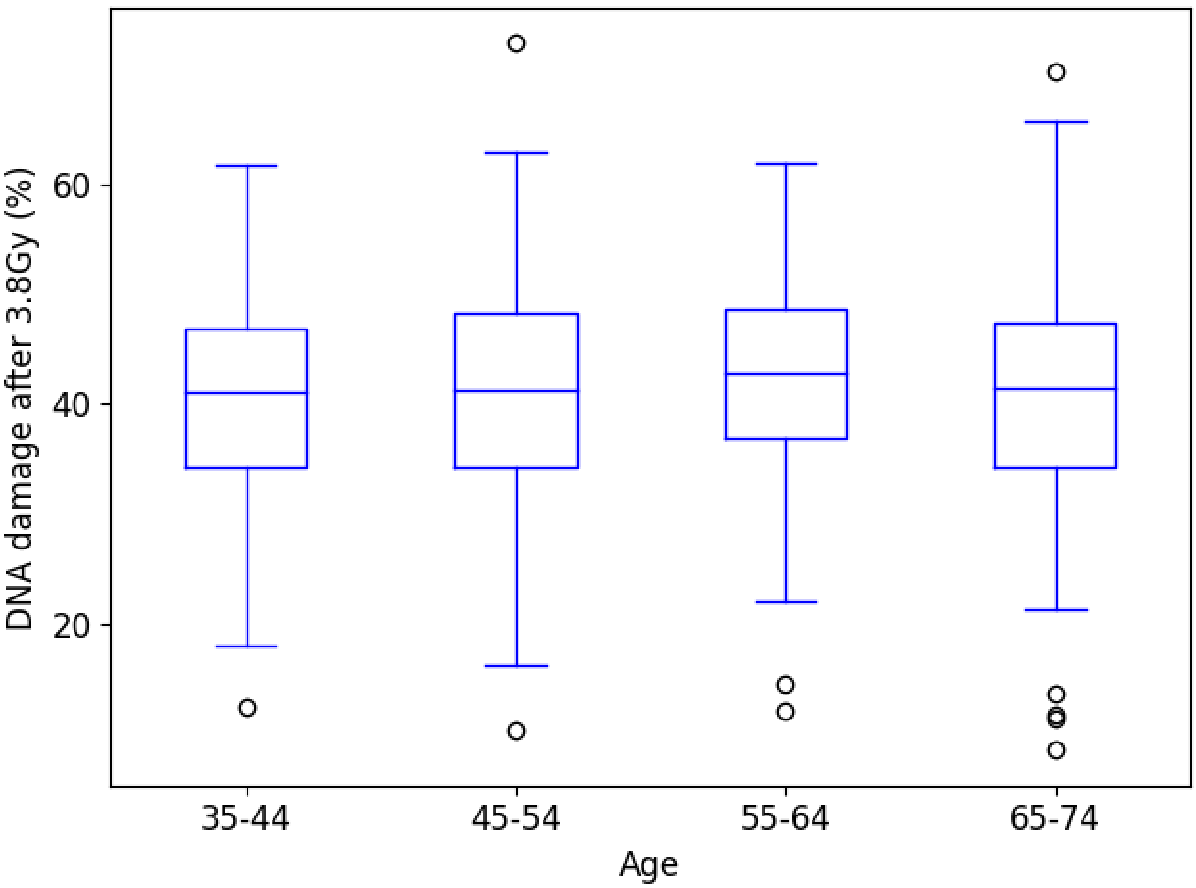

Figure S10. DNA damage after 3.8 Gy (%) values representation representation per age decades for males (635 subjects). The boxes show the median values, the 25th and 75th percentile. Whiskers indicate the 5th and 95th percentile. Outliers are displayed for each age-group.
